# Effects of Balance Training Using a Virtual Reality Program in Hemiplegic Patients

**DOI:** 10.3390/ijerph19052805

**Published:** 2022-02-28

**Authors:** Jung-Ah Kwon, Yoon-Kyum Shin, Deok-Ju Kim, Sung-Rae Cho

**Affiliations:** 1 Department of Occupational Therapy, Yonsei University College of Medicine, Seoul 03722, Korea; kjaot@yuhs.ac; 2 Department and Research Institute of Rehabilitation Medicine, Yonsei University College of Medicine, Seoul 03722, Korea; kyum309@yuhs.ac; 3 Brain Korea 21 PLUS Project for Medical Science, Yonsei University College of Medicine, Seoul 03722, Korea; 4 Department of Occupational Therapy, College of Health & Medical Sciences, Cheongju University, Cheongju 28503, Korea; 5 Graduate Program of Biomedical Engineering, Yonsei University College of Medicine, Seoul 03722, Korea; 6 Rehabilitation Institute of Neuromuscular Disease, Yonsei University College of Medicine, Seoul 03722, Korea

**Keywords:** Wii Fit balance training, hemiplegic patients, balance confidence, health-related quality of life

## Abstract

Therapeutic goals for hemiplegic patients with neurological impairments are mainly focused on improving their independent lives. Based on the previously reported effectiveness of Wii Fit balance training, this study investigated the most influential outcomes after long-term intensive training (including balance and functional factors) on quality of life in hemiplegic patients. The intervention group (n = 21) received Nintendo Wii Fit balance training under supervision, and control group (n = 20) received conventional balance training by an occupational therapist. Two groups were matched based on age and onset duration. Both groups received a total of 15 treatments for 30 min per session, twice a week for 8 weeks. There were significant improvements not only in balance confidence and activities of daily living, but also in body composition, such as fat proportion and metabolic rate, in the intervention group compared to the control group (*p* < 0.05). In particular, balance confidence significantly affected EuroQoL Visual Analogue Scale according to stepwise multiple regression analyses in this study. These results demonstrated that Wii Fit balance training using virtual reality improved the quality of life of hemiplegic patients while overcoming the asymmetrical weight distribution of the affected side via the self-modulating biofeedback exercises.

## 1. Introduction

Hemiplegic patients with neurological impairments suffer from their asymmetrical weight distributions in their lower extremities during dynamic daily activities [1,2,3]. Impaired balance affects their quality of life by reducing their confidence in their ability to perform normal activities in social settings [4]. As the neurologically damaged population increases, improving their health to support their social participation is becoming a critical issue in the rehabilitation field [5]. A sedentary lifestyle due to reduced mobility worsens their body composition, causing an increase in fat mass and a decrease in metabolic rate [6,7,8]. This problem could cause secondary complications, including obesity, in addition to their underlying conditions [9].

Self-controlled balance is necessary for daily activities, such as walking in dynamic environments [10]. Therefore, neurological patients should improve their balance via intensive long-term interventions [11]. However, a conventional therapy that includes mat activities for trunk and postural control has limitations, such as a lack of motivational aspects and failure to mimic useful activities in reality [12,13].

Among the beneficial interventions, Wii Fit balance training using game-based virtual reality has been reported to enhance balance and daily functional capacity based on biofeedback [14,15]. Indeed, some groups revealed that Wii Fit balance training is more effective than conventional treatment, resulting in enhanced functional capacity in activities such as walking [16,17]. Oliveira et al. (2015) revealed that Wii Fit games were superior in terms of improving balance and gait compared to similar training with no games in chronic stroke patients [18]. Da Silva Ribeiro et al. (2015) identified that virtual rehabilitation using the Nintendo Wii Fit increased physical functioning more when compared to conventional physical therapy in post-stroke hemiparetic patients [19]. Unibaso-Markaida and Iraurgi (2021) identified that Wii sports improved mobility and quality of life compared to a control group based on the effect size [20].

Other previous studies indicated that virtual reality-based exercise could improve quality of life, even assisting the recovery of mental health in neurologically impaired populations [21,22]. Cano-Manas et al. (2020) found that a video-game-based therapy with virtual environments was effective at improving quality of life, balance and function in patients with subacute stroke [23]. Manuli et al. (2020) also showed that robotic treatment with virtual reality positively influenced quality of life and psychological well-being in patients with chronic stroke [24]. Fundamentally, Wii Fit program contains various and interesting games to encourage postural control during exercises. The self-controlled posture and weight distribution demands could lead to improved confidence in independent dynamic activities [25,26].

Although these beneficial outcomes have been found with Wii Fit balance training, changes in body composition, including improvements in fat and muscle proportions, and metabolic rate, were not identified in hemiplegic patients with neurological impairments prior to this study. The present study is the first investigation to predict the most influential factor among balance and functional factors on quality of life. Therefore, we aimed to examine the effects of Wii Fit balance training for hemiplegic patients with neurological impairments on their balance, daily functions, body composition and quality of life.

## 2. Materials and Methods

### 2.1. Patients

Between 2018 and 2019, we selected 41 patients diagnosed with hemiplegia due to neurological impairments after reviewing medical records at Severance Hospital, Yonsei University College of Medicine in Seoul. This research was conducted with approval from the Institutional Review Board of Severance Hospital, Yonsei University (IRB Number 4-2019-0621). A total of 41 individuals aged from 19 to 65 years were finally included in this retrospective study. The inclusion criteria were as follows: the patient had onset duration more than 6 months after the brain injury, including stroke, traumatic brain injury (TBI), or brain tumor; the patient had to be an independent walker; and the patient had to have no problems with cognition. The exclusion criteria were as follows: unable to walk independently more than 30 m indoors; problems with visual perception; bilateral hemispheric or cerebellar lesions; previous use of Nintendo Wii Fit. Two groups were matched based on age and onset duration, retrospectively. The total number of subjects was 41, including 21 individuals in the intervention group (7 males and 14 females; mean age: 46.52 ± 14.15 years; onset duration: 9.16 ± 7.92 years) and 20 controls (12 males and 8 females; mean age: 47.00 ± 13.82 years; onset duration: 6.11 ± 4.60 years) (Table 1). Distributions of gender and diagnosis between groups were not significant by Chi-square test (*p* > 0.05). The authors estimated a sample size from a priori power analysis calculated with the effect size based on a pervious study [27]. There was a significant difference in balance parameters between the two groups (i.e., Wii Fit balance training and control groups) post-test (*d* = 0.98) with a statistical power of 0.80 (two-tailed test; α = 0.05). The analysis indicated that at least 36 individuals (eighteen per each group) would be needed to find the proper group difference.

### 2.2. Procedures

**Figure 1:**
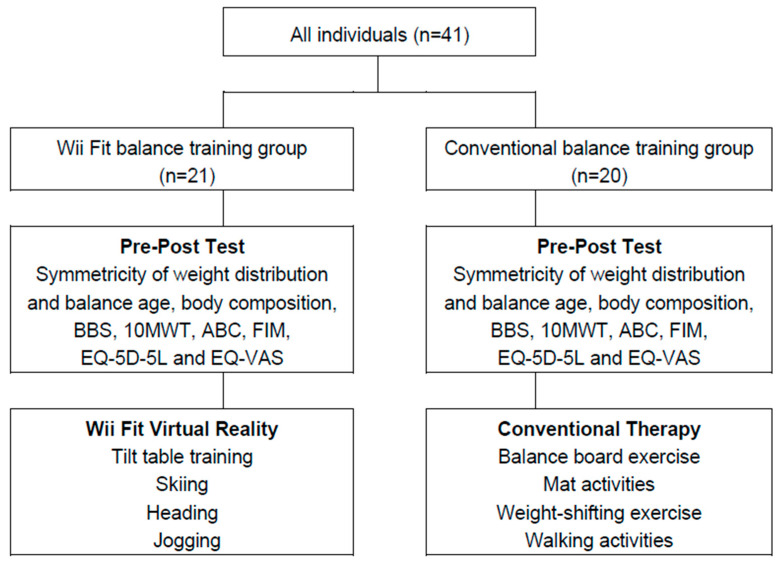
Procedures of the study.

Each session was conducted in a clinical room for occupational therapy. All individuals received a total of 15 sessions of balance training: Wii Fit training for the intervention group and conventional training for the control group, twice a week for 8 weeks. The authors used all outcomes from routine follow-up assessments: balance and walking functions, balance confidence, body composition, and quality of life, for which the patients regularly visited the hospital (Figure 1). In the control group, conventional balance training was conducted in each session for 30 min, including exercise on balance boards for trunk control, mat activities for proximal stabilization, weight-shifting exercises for equally distributing weight between both sides and walking activities with/without assistance for postural control. In the intervention group, the Wii Fit balance training was conducted on the Wii Balance Board in each session for 30 min, which included jogging and skiing (trying not to hit obstacles) for trunk and postural control in dynamic virtual reality environments, tilt table-balancing (trying to guide balls into the holes) for dynamic balance on an unstable surface, and heading (trying to hit a soccer ball with the head and escaping from other oncoming objects at the same time) for weight transfer and visual perception [28].

### 2.3. Outcome Measures

#### 2.3.1. Symmetricity of Weight Distribution and Balance Age

The Wii Balance Board (WBB; Nintendo, Kyoto, Japan) was utilized to assess static standing balance. The WBB is similar to a typical force platform that detects center of pressure via four electrical load sensors. Weight bearing asymmetry between the lower extremities was calculated as percentages of weight distribution on the two sides (50% each would indicate equal distribution in hemiplegic patients with neurological impairments in this study). The Wii Fit age was also calculated before and after intervention. Younger Wii Fit age means better balance. The authors used the WBB based on previous studies of its validity and reliability [29].

#### 2.3.2. Body Composition

Body composition using InBody (Biospace, California, CA, USA) was measured to determine body mass index (kg/m^2^) based on height and weight, fat mass and muscle mass (kg), body fat rate and metabolic rate (%). Individuals were measured while standing according to the manufacturer’s guidelines. The circumference (cm) of the mid-thigh at the center between the inguinal crease and proximal border of the patella was measured with a tape measure. The authors used this apparatus which is available nearly worldwide based on previous studies of its validity and reliability [30].

#### 2.3.3. Berg Balance Scale

The Berg balance scale (BBS) was utilized to measure an individual’s balance function. This scale consists of 14 items with total scores ranging from 0 to 56 scores (0 to 4 for each item). The authors utilized the BBS to assess changes in balance by the intervention, based on previous studies of its validity and reliability [31].

#### 2.3.4. Walking Speed

Walking speed (m/s) using the 10 m walk test (10MWT) was measured in a clinical room. Regular walking speed was measured at a comfortable speed, and fast walking speed was measured at the fastest gait speed possible on a 10-m walkway. An average value of three trials was recorded for an individual in this study [32]. The authors utilized 10MWT to assess changes in locomotive function by the intervention based on previous studies of its validity and reliability [33,34].

#### 2.3.5. Balance Confidence

Balance confidence was measured using the activity-specific balance confidence (ABC) when individuals performed activities. It consists of a 16 item self-reported questionnaire with scores ranging from 0 to 100, indicating no confidence to complete confidence when performing the activity. The ABC suggests that scores lower than 50 indicate low functional levels, scores between 50 to 80 are indicative of medium functional levels and scores above 80 indicate high functional levels. The authors utilized the ABC to assess changes in balance confidence by the intervention based on previous studies of its validity and reliability [35].

#### 2.3.6. Activities of Daily Living

Activities of daily living (ADL) was measured using the functional independence measure (FIM) to evaluate the patient’s performance of daily functions. The test consists of 18 items with possible scores ranging from 18 to 126 overall (1 to 7 for each item). Among 18 items, the main five items were included to identify personal mobility, including transfers to or from (a bed/chair/wheelchair, a toilet, and a tub/shower) and locomotion (walking/wheelchair, and stairs), giving a total score of 35 [36]. The authors utilized FIM based on previous studies of its validity and reliability [37].

#### 2.3.7. Quality of Life

The health-related quality of life was assessed using EuroQoL 5 Dimensions (EQ-5D-5L) and EuroQoL Visual Analogue Scale (EQ-VAS). EQ-5D-5L consists of five domains, including mobility, self-care, usual activities, pain/discomfort and anxiety/depression. It allows five levels of response from 1 to 5 (no problems to extreme problems). EQ-VAS is a visual analogue measure with scores ranging from 0 to 100 (the worst health to the best health) based on personal opinion [38]. The authors utilized these tests to assess changes in quality of life based on previous studies of their validity and reliability [39].

### 2.4. Statistical Analyses

Statistical analyses were performed using the Statistical Package for Social Sciences (SPSS) version 21.0. (IBM, Chicago, IL, USA) A Chi-square test was used to test the homogeneity of subjects in two groups. A Shapiro–Wilk test was used to analyze the normality of the variables and to support reasonable use of a parametric test. This normality test demonstrated that all variables had normal distributions in this study (*p* > 0.05). For pre- and post-test comparisons, paired *t*-tests were performed, and independent *t*-tests were used to compare differences in variables between the two groups. In the independent *t*-tests, general characteristics at baseline and changes in outcome data relative to pre-test (Δ) were compared between the two groups. Stepwise multiple regression analyses were used to identify factors affecting health-related quality of life. Statistical significance was set to *p* < 0.05.

## 3. Result

### 3.1. Symmetricity of Weight Distribution and Balance Age

**Figure 2:**
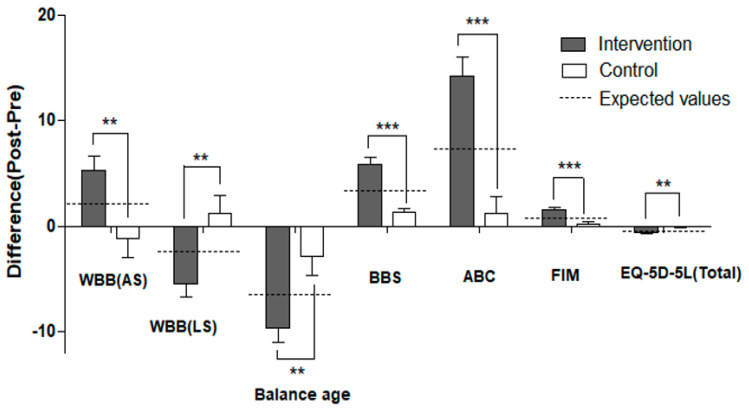
Representative outcomes showing significant group differences in this study. Values are mean ± SE; ** *p* < 0.01, *** *p* < 0.001; Dotted line: the expected value indicating an average of all variables from individuals in each outcome measure; WBB (AS/LS): weight distribution using Wii Balance Board in the affected side or the less affected side; BBS: Berg Balance Scale; ABC: Activities-specific Balance Confidence scale; FIM: Functional Independence Measure; EQ-5D-5L: EuroQoL 5 Dimensions (Total score).

The intervention group showed enhanced symmetricity of weight distribution between the lower extremities (more weight on the affected side) following the Wii Fit balance training (*p* < 0.01), indicating improved balance control for the hemiplegic patients with neurological impairments. The intervention group also showed significantly younger Wii Fit age following the Wii Fit balance training (*p* < 0.001) (Table 2 and Figure 2).

### 3.2. Balance and Functional Outcomes

Both groups showed statistically significantly improved balance according to the BBS (*p* < 0.01). However, balance confidence tested by the ABC (*p* < 0.001); ADL tested by the FIM (*p* < 0.001); and regular walking speed (*p* < 0.001) and fast walking speed (*p* < 0.01) tested by 10MWT were statistically significantly improved in Wii Fit balance training group. Differences between the two groups were significant in balance function according to the BBS (*p* < 0.001), balance confidence tested by the ABC (*p* < 0.001) and ADL tested by the FIM (*p* < 0.001) (Table 3 and Figure 2).

### 3.3. Body Composition

After balance training, the intervention group showed significant changes in body fat proportion, body fat amount, muscle mass and basic metabolic rate (*p* < 0.05). However, there were no significant changes in body composition in the control group. Differences between the two groups were significant for body fat amount and basic metabolic rate (*p* < 0.05). In addition, both groups showed changes in the circumference of the mid-thigh, though only in the affected side (*p* < 0.05). However, the difference in mid-thigh circumference between the two groups was not significant (Table 4 and Figure 2).

### 3.4. Quality of Life

The intervention group showed significant changes in the mobility domain and total score of EQ-5D-5L after Wii Fit balance training (*p* < 0.01). However, there were no significant changes in the control group. The difference between the two groups in the total score was significant (*p* < 0.01). The intervention group also showed significant changes in EQ-VAS (*p* < 0.01). However, the control group did not show significant changes for this scale. Differences between the two groups were not found for EQ-VAS (Table 5 and Figure 2).

### 3.5. Factors Affecting Health-Related Quality of Life

In stepwise multiple regression analyses, to identify the most influential factor on quality of life, two regression models were presented. There were no multicollinearity in the model 1 (the range of the tolerance level, 0.48–0.94 and the variance inflation factor, 1.05–2.09), and in the model 2 (0.23–0.78 and 1.07–4.24). The regression analysis of the first model revealed diagnostic factors, including stroke (β = −0.650, *p* = 0.002) and TBI had significant effects on EQ-VAS. The regression analysis of the final model, including functional factors, demonstrated that ABC (β = 0.411, *p* = 0.017) was the most influential factor on EQ-VAS, indicating increased balance confidence provided improved quality of life (Table 6).

## 4. Discussion

In this study, the authors investigated the effects of Wii Fit balance training using virtual reality on the body compositions and health-related quality of life of chronic hemiplegic patients. The aim was to see functional improvements improve those metrics. Significantly better balance from enhanced symmetricity, and consequently better performance of daily activities, affected quality of life. Improved metabolic function was also achieved by the Wii Fit balance training.

Specifically, the intervention group showed significant improvements in balance, including static and walking balance (i.e., BBS, *d* = 8.51 between two groups (Δ) with an actual power of 0.97). Enhanced confidence and better performance of activities of daily living were also achieved. For hemiplegic patients, the balance training focused on improving symmetricity between the lower extremities through dynamic virtual reality programs. Previous studies have reported that improved dynamic balance is closely associated with the performance of functional activities, including walking [13,18]. By using biofeedback and relying on self-motivated training, symmetricity was improved, and eventually controlled mobility was as well, which increased walking speed in comparison to the patients who received conventional therapy under supervision by therapists [12,19,20]. From a variety of previous studies, exercises in virtual reality environments using biofeedback have been known to be effective at improving balance and walking in neurological patients, such as stroke patients [11,15,16,17,21,40].

As novel parameters, changes in body composition, including fat mass and muscle mass, were measured after training in this study, which aimed to better explain any improvements in health from the Wii Fit balance training. We found significant accumulative effects from intensive long-term training over 8 weeks: improvements in fat mass and muscle mass, and increased energy expenditure. Although changes in the mid-thigh circumference were found in both groups, these changes coincided with changes in fat/muscle composition only in the Wii Fit balance training group. These beneficial changes suggest it is an effective therapy to improve reduced metabolic function in chronic neurological patients with limited activity [41].

As a self-modulating exercise in a variety of weight-bearing directions, the Wii Fit balance training was applied to control weight distribution in hemiplegic patients. Enhanced muscle activation during sufficient weight loading on the affected lower extremity eventually resulted in improved balance, performance of daily activities and body composition [12,18,20,41].

Improved balance significantly affected quality of life after Wii Fit balance training in hemiplegic patients with neurological impairments. Balance confidence was the most influential factor on quality of life in relation to health. Based on the regression analysis, quality of life was actually affected most by the balance confidence during dynamic activities. Although there was no statistical significance, the anxiety and depression score was reduced in the intervention group in contrast to the control group. This suggests that an increase in the balance confidence during activities is an important therapeutic focus for hemiplegic patients [42].

Taken together, the results of this study suggest that self-controlled mobility exercises using Wii Fit balance training constitute a beneficial therapeutic approach that can improve the performance of daily activities via confidence and better balance, thereby improving quality of life in chronic hemiplegic patients with neurological impairments.

## 5. Conclusions

This study found that self-modulating Wii Fit balance training with biofeedback enhanced balance confidence, thereby improving quality of life, in hemiplegic patients with neurological impairments. Although the results of this study indicate beneficial changes in body composition, such effects should be identified whether it continue for many years. For this, the virtual reality program should be used at the participants’ homes as alternatives to face-to-face sessions in an extended future study. Through these further studies, the Wii Fit balance training program may prove able to sustain the beneficial effects we observed in chronic hemiplegic patients.

## Figures and Tables

**Table 1:** Clinical characteristics of subjects (N = 41).

	Intervention Group (n = 21)	Control Group (n = 20)	*p*
Age, years	46.52 ± 14.15 ^†^	47.00 ± 13.82	0.252
Gender, n(%)			0.087
Male	7 (33.3)	12 (60.0)	
Female	14 (66.7)	8 (40.0)	
Diagnosis, n (%)			0.511
Stroke	15 (71.4)	14 (70.0)	
Traumatic brain injury	2 (9.5)	4 (20.0)	
Tumor	4 (19.1)	2 (10.0)	
Onset duration, years	9.16 ± 7.92	6.11 ± 4.60	0.452
Affected side, n (%)			0.867
Rt. side	11 (52.3)	11 (55.0)	
Lt. side	10 (47.7)	9 (45.0)	

^†^ Mean ± SD; continuous variables analyzed by independent *t*-test; categorical variables analyzed by Chi-square test.

**Table 2:** Changes in the symmetricity of weight distribution and balance age.

Variables (Unit) Group	Pre	Post	*p* ^a^	Difference	*p* ^b^
Affected side (%)					
Intervention	44.83 ± 7.25 ^†^	50.25 ± 4.36	0.001 **	5.31 ± 1.33 ^§^	0.004 **
Control	51.26 ± 7.67	50.05 ± 5.24	0.492	−1.21 ± 1.72	
Less affected side (%)					
Intervention	55.17 ± 7.25	49.75 ± 4.36	0.001 **	−5.42 ± 1.33	0.004 **
Control	48.75 ± 7.67	49.95 ± 5.24	0.492	1.21 ± 1.72	
Balance age (years)					
Intervention	59.14 ± 12.95	49.43 ± 11.50	0.000 ***	−9.71 ± 1.32	0.003 **
Control	58.20 ± 15.04	55.15 ± 16.17	0.108	−2.90 ± 1.75	

^†^ Mean ± SD; ^§^ Mean ± SE; ** *p* < 0.01 *** *p* < 0.001; *p* ^a^, paired *t*-test; *p* ^b^, independent *t*-test.

**Table 3:** Changes in balance and functional outcomes.

Variables (Unit) Group		Pre	Post	*p* ^a^	Difference	*p* ^b^
BBS (score)						
Intervention		42.10 ± 9.36 ^†^	48.10 ± 7.18	0.000 ***	5.90 ± 0.69 ^§^	0.000 ***
Control		47.00 ± 8.52	48.35 ± 7.71	0.001**	1.30 ± 0.33	
ABC (score)						
Intervention		55.95 ± 22.74	69.76 ± 20.98	0.000 ***	14.19 ± 1.82	0.000 ***
Control		64.99 ± 29.82	66.10 ± 27.87	0.501	1.16 ± 1.64	
FIM (score)						
Intervention		30.14 ± 2.35	31.67 ± 2.80	0.000 ***	1.52 ± 0.26	0.000 ***
Control		30.90 ± 3.34	31.15 ± 3.01	0.096	0.25 ± 0.14	
Walking speed (m/s)	Regular speed					
	Intervention	1.54 ± 0.50	1.29 ± 0.41	0.000 ***	−0.22 ± 0.05	0.091
	Control	1.30 ± 0.51	1.25 ± 0.54	0.390	−0.08 ± 0.07	
	Fast speed					
	Intervention	1.21 ± 0.50	1.06 ± 0.40	0.003 **	−0.14 ± 0.05	0.144
	Control	1.03 ± 0.38	0.96 ± 0.32	0.219	0.05 ± 0.12	

^†^ Mean ± SD; ^§^ Mean ± SE; ** *p* < 0.01; *** *p* < 0.001; *p* ^a^, paired *t*-test; *p* ^b^, independent *t*-test; BBS: Berg Balance Scale; ABC: Activity-specific Balance Confidence scale; FIM: Functional Independence Measure; walking speed in the 10-meter walk test.

**Table 4:** Changes in body composition.

Variable (Unit) Group		Pre	Post	*p* ^a^	Difference	*p* ^b^
Body composition	BMI (kg/m^2^)					
	Intervention	23.32 ± 2.89 ^†^	23.44 ± 3.29	0.666	0.12 ± 0.27 ^§^	0.842
	Control	24.89 ± 3.39	24.83 ± 3.58	0.849	0.04 ± 0.29	
	Weight (kg)					
	Intervention	64.73 ± 11.65	64.30 ± 12.41	0.473	−0.42 ± 0.59	0.169
	Control	69.79 ± 14.34	70.51 ± 14.95	0.213	0.72 ± 0.56	
	Body fat rate (%)					
	Intervention	33.22 ± 7.60	29.98 ± 8.06	0.034 *	−3.30 ± 1.41	0.109
	Control	29.99 ± 7.74	29.55 ± 7.32	0.571	−0.70 ± 0.71	
	Body fat amount (kg)					
	Intervention	21.09 ± 5.39	18.40 ± 4.53	0.021 *	−2.69 ± 1.08	0.021 *
	Control	21.04 ± 6.66	21.39 ± 7.11	0.600	0.35 ± 0.65	
	Muscle mass (kg)					
	Intervention	23.37 ± 6.30	24.99 ± 8.17	0.039 *	1.61 ± 0.73	0.122
	Control	26.83 ± 6.82	27.25 ± 6.76	0.133	0.37 ± 0.27	
	Basic metabolic rate (%)					
	Intervention	1303.29 ± 232.26	1374.24 ± 256.43	0.017 *	70.95 ± 27.18	0.038 *
	Control	1438.25 ± 268.22	1437.50 ± 240.49	0.969	−0.75 ± 18.91	
Mid-thigh circumference	Affected side (cm)					
	Intervention	52.64 ± 4.91 ^†^	54.33 ± 4.41	0.027 *	1.71 ± 0.70 ^§^	0.658
	Control	49.95 ± 8.94	51.25 ± 9.31	0.021 *	1.33 ± 0.51	
	Less affected side (cm)					
	Intervention	54.88 ± 4.34	56.48 ± 4.56	0.065	1.60 ± 0.82	0.249
	Control	52.65 ± 7.82	53.18 ± 8.24	0.305	0.48 ± 0.50	

^†^ Mean ± SD; ^§^ Mean ± SE; * *p* < 0.05; *p* ^a^, paired *t*-test; *p* ^b^, independent *t*-test; BMI: Body Mass Index.

**Table 5:** Changes in quality of life: EQ-5D-5L and EQ-VAS.

Variables (Unit) Group		Pre	Post	*p* ^a^	Difference	*p* ^b^
EQ-5D-5L	Mobility (score)					
	Intervention	2.33 ± 0.91 ^†^	1.81 ± 0.87	0.001 **	−0.43 ± 0.18 ^§^	0.051
	Control	2.20 ± 0.95	2.15 ± 0.93	0.330	−0.05 ± 0.05	
	Self-care (score)					
	Intervention	1.95 ± 0.80	1.95 ± 0.80	—	—	—
	Control	1.95 ± 0.69	1.95 ± 0.69	—	—	
	Activity (score)					
	Intervention	2.33 ± 0.73	2.33 ± 0.73	—	—	—
	Control	2.20 ± 0.62	2.20 ± 0.62	—	—	
	Pain (score)					
	Intervention	2.10 ± 0.70	2.10 ± 0.70	—	—	—
	Control	2.20 ± 0.70	2.20 ± 0.70	—	—	
	Anxiety (score)					
	Intervention	1.95 ± 0.67	1.90 ± 0.62	0.329	−0.05 ± 0.05	0.329
	Control	1.95 ± 0.76	1.95 ± 0.76	—	—	
	Total score					
	Intervention	10.67 ± 2.33	10.10 ± 2.34	0.001 **	−0.57 ± 0.15	0.003 **
	Control	10.50 ± 2.98	10.45 ± 2.96	0.330	−0.05 ± 0.05	
EQ-VAS (score)	Intervention	76.24 ± 14.07 ^†^	80.90 ± 10.30	0.003 **	2.76 ± 1.62 ^§^	0.592
	Control	75.45 ± 16.89	77.30 ± 14.86	0.110	1.70 ± 1.08	

^†^ Mean ± SD; ^§^ Mean ± SE; ** *p* < 0.01; —, not observed difference between variables; *p* ^a^, paired *t*-test; *p* ^b^, independent *t*-test; EQ-5D-5L: EuroQoL 5 Dimensions; EQ-VAS: EuroQoL Visual Analogue Scale.

**Table 6:** Factors affecting health-related quality of life.

Variable (Reference)	Model 1				Model 2			
	β	*p*	Odds Ratio	95% CI	β	*p*	Odds Ratio	95% CI
Gender (female)	0.105	0.479	2.651	−4.878–10.180	0.202	0.203	5.078	−2.885–13.041
Age	−0.030	0.870	−0.027	−0.364–0.309	0.113	0.545	0.104	−0.242–0.449
Diagnosis (tumor)								
Stroke	−0.650	0.002 **	−17.927	−28.828–−7.026	−0.448	0.021 *	−13.457	−24.775–−2.139
TBI	−0.431	0.046 *	−15.281	−30.251–−0.310	−0.377	0.074	−13.382	−28.150–1.387
Onset duration	−0.217	0.150	−0.417	−0.991–0.158	−0.185	0.202	−0.355	−0.910–0.200
BBS					0.307	0.070	0.530	−0.046–1.107
ABC					0.411	0.017 *	0.214	0.040–0.388
FIM					0.273	0.100	1.205	−0.243–2.652
R^2^	0.279				0.396			
Adjusted R^2^	0.176				0.245			
F(*p*)	2.771 (0.036 *)				2.624 (0.025 *)			

* *p* < 0.05 ** *p* < 0.01; TBI: Traumatic Brain Injury; BBS: Berg Balance Scale; ABC: Activity-specific Balance Confidence scale; FIM: Functional Independence Measure; CI: Confidence Interval.

## Data Availability

Not applicable.
